# Cross-Cultural Adaptation and Validation of the Arabic Version of the Physical Activity Scale for Individuals with Physical Disabilities in Saudi Arabia (PASIPD-AR)

**DOI:** 10.3390/healthcare12020179

**Published:** 2024-01-11

**Authors:** Majed M. Alhumaid, Mohamed A. Said, Yuhanis Adnan, Selina Khoo

**Affiliations:** 1 Department of Physical Education, College of Education, King Faisal University, Al-Ahsa 31982, Saudi Arabia; 2 Faculty of Sports and Exercise Science, Universiti Malaya, Kuala Lumpur 50603, Malaysia

**Keywords:** physical activity, health, physical disability, reliability, translation, validation, scale

## Abstract

This study aimed to cross-culturally adapt and validate the Arabic version of the Physical Activity Scale for Individuals with Physical Disabilities (PASIPD) with Saudi Arabian participants. The study encompassed four distinct stages: (i) translation and subsequent back-translation; (ii) a preliminary assessment aimed at evaluating the quality of the translated scale; (iii) an assessment of the reliability of the measures employed; and (iv) a comprehensive examination of the validity of the measures. A sample of Saudi Arabian participants with physical disabilities (*N =* 206) took part, ranging in age from 18 to 70 years old, with an average age of 39.56 years and a standard deviation of 12.16. The findings obtained from the reliability tests indicated a notable level of internal consistency and stability. Experts and confirmatory factor analysis were employed to establish the face, content, and construct validity. The findings of the assessment of the Arabic version of PASIPD demonstrated a satisfactory degree of reliability and validity, rendering it suitable for implementation within the Saudi Arabian setting.

## 1. Introduction

The manifestation of disability is contingent upon the interplay between various health disorders, such as dementia, blindness, or spinal cord damage, and various environmental and personal circumstances. Currently, approximately 1.3 billion individuals, accounting for 16% of the global population, are experiencing significant impairments [1]; this trend is increasing due to better treatments for noncommunicable diseases, which have extended life expectancy worldwide [2]. That said, individuals with physical disabilities can expect a reduced life expectancy, inferior health outcomes, and encounter greater challenges in their daily activities compared to those without disabilities [2].

Physical disability is a prominent form of disability observed in both children and adults [3]. According to Okoro et al. [4], in 2016, around 61.4 million (25.7%) of noninstitutionalized individuals in the United States had some form of physical disability. The most common impairments were physical (13.7%), followed by cognitive (10.8%), independent living (6.8%), hearing (5.9%), vision (4.6%), and self-care (3.7%). Recent statistical data shows that approximately 7.1% of Saudi Arabians have a disability, of which 3.9% are physical disabilities. These rates are anticipated to rise due to the ongoing escalation in health risk factors that include but are not limited to obesity, physical inactivity, traffic accidents, and chronic diseases [3].

According to the Americans with Disabilities Act [5], a physical disability is a substantial and long-term condition affecting a part of a person’s body that impairs and limits their physical functioning, mobility, stamina, or dexterity. This definition encompasses a range of conditions including cerebral palsy, stroke, spina bifida, arthritis, spinal cord injury, epilepsy, and muscular dystrophy. Individuals with physical disabilities may face challenges when engaging in routine tasks like ambulation, maintaining an upright position, assuming a seated posture, manipulating their upper extremities, and coordinating muscular movements. Nevertheless, individuals with physical disabilities can benefit in a variety of ways from participating in physical activity (PA). Overall health, as well as physical strength and mobility, can be improved by regular exercise.

According to World Health Organization (WHO) [6], engaging in PA can be effective in helping people manage their weight, enhance their mental well-being, and reduce the likelihood of premature mortality, cardiovascular diseases, type 2 diabetes, and certain forms of cancer. Additionally, engaging in PA has positive effects on mental health, including the reduction in symptoms associated with depression and anxiety, as well as the enhancement of social standing [7]. For example, Arbour-Nicitopoulos et al. [8] discovered that individuals without disabilities exhibited a more positive perception of individuals with disabilities who engaged in PA compared to those who did not engage in PA. The study found that individuals with disabilities who engaged in regular exercise were perceived to possess higher levels of friendliness, self-reliance, and persistence, as well as better overall health and fitness in comparison to both non-exercisers and individuals without disabilities in the control groups. Hence, the involvement of individuals with disabilities in PA appears to mitigate prejudices held by non-disabled individuals towards the former, thereby reducing the marginalization of those with disabilities as posited by the social model of disability [9]. Anderson and Heyne [10] and Orr et al. [9] proposed that engaging in PA holds “amplified importance” for individuals with disabilities as it can help address cognitive issues such as attention deficit disorder, emotional challenges including depression and low self-esteem and self-efficacy, and social difficulties such as loneliness. These disorders are common among individuals with disabilities, making PA especially crucial in the prevention and mitigation of such conditions.

Adults with disabilities should engage in 150–300 min of moderate-intensity aerobic PA, 75–150 min of vigorous-intensity aerobic PA, or an equivalent combination of both per week [2]. Furthermore, it is recommended that people with disabilities engage in moderate to high-intensity muscle-strengthening activities involving all major muscle groups at least twice a week. These exercises have additional health advantages. The WHO also recommends that older people engage in a variety of multi-component PA on a weekly basis. These exercises should be performed at a moderate to high intensity, with a focus on functional balance and strength training. It is recommended that these activities be performed three or more days per week to boost functional ability and reduce the risk of falling. According to the Centers for Disease Control and Prevention [11], it is critical to seek advice from a healthcare professional or PA specialist to gain a thorough understanding of how a disability or medical condition may affect one’s ability to engage in PA safely. Walking allows most individuals, including people with disabilities (who can ambulate or move around with the use of assistive devices such as walkers) to participate in a PA way of life [7]. Walking is the most prevalent type of PA reported by active adults with mobility constraints [12].

Hence, it is imperative to ascertain the degree of involvement in PA of individuals with physical disabilities for three main reasons. Firstly, it enables experts in the healthcare and research domains to assess the effectiveness of rehabilitation programs and therapies. Tracking an individual’s activity levels can provide valuable data on their progress and enable treatment adjustments. Secondly, it enables individuals to effectively track their PA levels, set personal goals, and make informed health choices based on accurate data. Thirdly, the evaluation of PA among individuals with disabilities can enhance our understanding of the associations between levels of exercise and health-related outcomes.

Accelerometry and pedometers are often employed to objectively quantify levels of PA. Regrettably, the cost of using such devices is often prohibitive in large-scale studies [13]. Observation-based approaches are also too expensive and impractical for large-scale investigations as they require comprehensive and accurate records to be kept by highly experienced observers [14]. Self-report surveys (i.e., questionnaires, interviews, and daily activity diaries) are commonly employed in comprehensive epidemiological investigations [13]. Self-report methods offer several advantages for researchers. Firstly, they are non-intrusive and do not influence the behavior of the individuals being studied. Secondly, these methods enable the assessment of multiple variables using a single instrument, such as leisure or professional activity, as well as the duration, intensity, and frequency of activity, along with estimated energy expenditure. Thirdly, self-report methods are straightforward to administer and score. Lastly, they are cost-effective [15]. The effectiveness of a self-report questionnaire relies on its ability to distinguish between recent acts and habitual behaviors [16]. The process of recalling recent events, such as activities conducted over the preceding days, may not provide an accurate representation of the usual pattern or routine of the individual in question. Long-term memory retrieval, such as the ability to recall activity patterns over the period of a year, could offer a more practical approach to uncovering these patterns. Nevertheless, it is crucial to acknowledge that the probability of inaccuracies in recollecting events escalates when such broad temporal frameworks are used [15]. In their systematic review, Doma et al. [17] found that the use of recall periods, such as the standard seven-day period, within the past twelve months, or within the previous seven days, is a frequently utilized approach in adult studies. This method shows a strong correlation with direct measurements of PA, such as accelerometers.

The Physical Activity Scale for People with Physical Disabilities (PASIPD) [18] is the most common seven-day PA recall instrument used to assess PA levels among individuals with physical disabilities. The PASIPD is a 13-question instrument that requests information about the following 3 subscales: (i) leisure time activities, (ii) household tasks, and (iii) occupational activity over the past week. Each scale item assesses the number of days and average hours per day of PA participation at different intensities. The scoring of the scale reflects a composite PASIPD score computed by multiplying the average hours per day by a MET value. The MET value is based on activity intensity and eventually expresses the PA patterns as MET-h/day [18]. The PASIPD had test–retest reliability and criterion validity comparable to well-established self-report PA questionnaires used in the general population [19]. Tanhoffer et al. [20] found no statistically significant difference between the two methods when they compared the energy expenditure estimated by PASIPD to that determined using a combination of doubly labeled water and indirect calorimetry. Total daily energy use was 1% overstated while PA energy expenditure was 3% underestimated by PASIPD. Lankhorst et al. [21], in their systematic review of the characteristics of instruments used to assess PA in wheelchair users, concluded that the PASIPD has received significant attention and shows promise as a self-report tool for assessing PA in manual wheelchair users. Additionally, the Physical Activity Recall Assessment for People with Spinal Cord Injury (PARA-SCI) was identified as a suitable instrument for evaluating PA levels specifically in manual wheelchair users with spinal cord injuries. Using PASIPD enables individuals to set achievable goals, monitor their progress, and make informed decisions about their health. In addition, this tool facilitates the development of personalized interventions and techniques to encourage PA among people with physical disabilities, ultimately helping to improve their overall health, level of independence, and quality of life.

Unfortunately, the Arab region, and Saudi Arabia in particular, suffers from a severe shortage of tools to assess the extent to which people with physical disabilities engage in PA. The persistence of this gap has hampered efforts to encourage physically active lifestyles and adapt care to the specific needs of this population. Targeted programs, effective resource allocation, and impact evaluation of initiatives to increase PA levels among people with disabilities are hampered by a lack of referral measures [22].

In recent years, the Saudi government has demonstrated an increased focus on promoting regular engagement in PA among its population. As part of its objectives for improving Saudi Arabia, the Vision 2030 program seeks to enhance the quality of life of its citizens. In terms of encouraging participation in PA and sport, its primary objective is to attain a participation rate of 40% by 2030. The Saudi General Statistics Authority reported a PA rate of 20.04% in 2019 and 29.7% in 2021, indicating a significant increase. Regrettably, the figures exclude those with disabilities due to the absence of culturally appropriate instruments in Saudi Arabia for assessing and ascertaining the PA levels of this demographic. Indeed, investigating the PA levels of individuals with physical disabilities will significantly contribute to the formulation of suitable strategies and initiatives aimed at attaining the desired PA goals for Saudi Arabians with disabilities. As a result, there is an urgent need to develop an Arabic-language instrument to facilitate the execution of this operation. The purpose of this study was to adapt the PASIPD cross-culturally into Arabic language (PASIPD-AR) and evaluate its psychometric properties among individuals with physical disabilities in Saudi Arabia.

## 2. Materials and Methods

**Figure 1:**
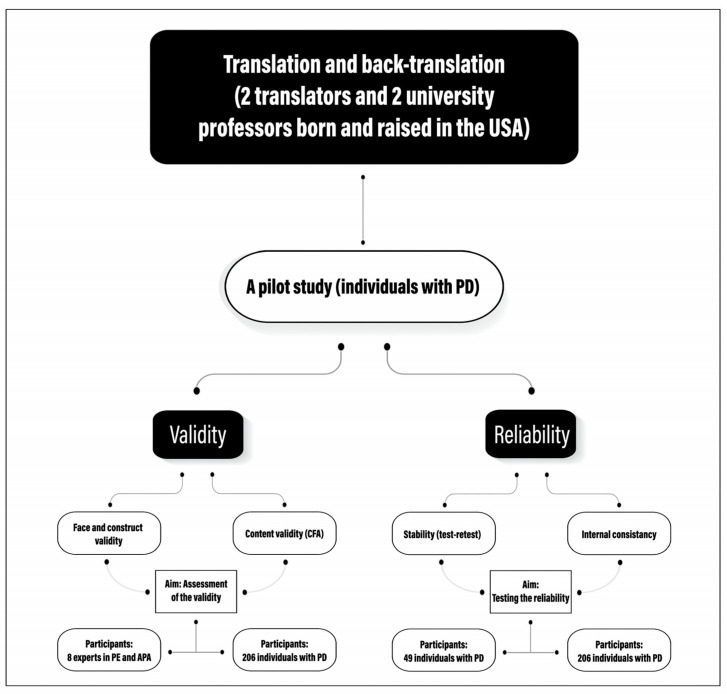
Graphical representation of data collection and analysis method for translation, validation, and reliability testing of the PASIPD-AR. CFA—confirmatory factor analysis; PD—physical disabilities; PE—physical education; APA—adapted physical activity.

The study was conducted between June 2023 and August 2023, following the guidelines of the Helsinki Declaration. It received approval from the Research Ethics Committee at King Faisal University (KFU-REC-2023-JUN-ETHICS1091). Each participant received a written informed consent form, which they reviewed and signed. This investigation involved multiple tests carried out in multiple steps, following an approach similar to that of Safipour et al. [23] and in consensus with the Consensus-based Standards for the selection of health status Measurement Instruments (COSMIN) study design checklist [24]. Figure 1 provides a summary of the study’s methodology and the approaches that were utilized.

### 2.1. Participants in the Main Study

Safipour et al. [23] suggested that a suitable sample size for factor analysis is from 200–300 participants. To gather the sample, participants with physical disabilities were randomly selected from three provinces within Saudi Arabia (Eastern province, Western province, and Riyadh province). The inclusion criteria were individuals with physical disabilities living in Saudi Arabia, whose native language is Arabic, and who are 18 years old and above. Given the hypothetical scenario of an equitable distribution of individuals with physical disabilities across different geographical areas, a sample size of 70 participants, allowing for a 20% attrition rate [25], was randomly selected from each province from the population of individuals receiving care and rehabilitation at institutions catering to those with disabilities. The determination of the sample size was conducted utilizing Cochran’s method, incorporating a margin of error of 5%, a Z-value of 1.96, and a proportion of 3.9% denoting the prevalence of physical disabilities within the Saudi population [3]. The PASIPD-AR was disseminated using three primary channels of communication: telephone, email, or in-person interviews conducted at a prearranged site. Participants who received dual invitations (e.g., via telephone and email) were instructed to respond to the questionnaire only once and to disregard the second invitation.

After analyzing the responses, a total of 206 respondents were selected and included in the subsequent analysis. The sample consisted of 72 female participants, accounting for 35% of the total, and 134 male participants, making up 65% of the total. The age range of the participants was between 18 and 70 years old, with a mean age of 39.56 years old and a standard deviation of 12.164 years. Out of all the participants, 42 individuals did not require any mobility aids. However, 20 participants used a cane, 120 relied on a wheelchair, and 24 utilized crutches to meet their mobility needs. Moreover, among the total sample, 139 individuals (67.5%) reported being in good health, 26 (12.6%) reported poor health, and 41 (19.9%) claimed to have excellent health. Additionally, 61 participants (29.6%) indicated a complete lack of PA, while 128 participants (62.1%) reported engaging in moderate levels of activity. Furthermore, 17 participants (8.3%) reported being active or highly active.

### 2.2. Physical Activity Scale for Individuals with Physical Disabilities (PASIPD)

The PASIPD is a modification of the Physical Activity Scale for the Elderly (PASE), which was adapted to be suitable for those with physical disabilities by Washburn et al. [18]. It consists of a total of 13 items, divided into three categories: (i) leisure time activities, (ii) household tasks, and (iii) occupational activity. The leisure time activities category comprises six items, including activities such as walking, wheeling outside the home, and engaging in exercise with light to moderate or strenuous sports for recreational purposes. The household tasks category also includes six items, covering activities such as light and heavy housework, outdoor gardening, carrying out repairs, and caring for another person. Lastly, the occupational activity encompasses one item. The first item is not assessed; instead, it serves as a guide, and the remaining 12 items enable the identification of five factors: Factor 1: home repairs, lawn mowing, and garden work (items 9, 10, 11); Factor 2: housework (items 7, 8, 12); Factor 3: vigorous sport and recreational activity (items 5, 6); Factor 4: light and moderate sport and recreational activity (items 3, 4); and Factor 5: occupational and transportation activities (items 2, 13). For all items, the participant is asked to recall the number of days in the past 7 days when they undertook these activities: never/seldom (1–2 days/week), sometimes (3–4 days/week), or often (5–7 days/week), and on average how many hours a day they participated (<1 h, 1 but <2 h, 2–4 h, >4 h).). For the occupational item, the hours per day are as follows: <1 h, 1 but <4 h, 5 but <8 h, ≥8 h. The PASIPD rating is calculated by multiplying the average daily hours spent on each activity by the metabolic equivalent (MET) associated with its intensity. The scores range from 0.0 MET h/day (indicating no activities performed) to 199.5 MET h/day (representing all listed activities performed for the maximum duration of days and hours) [18].

### 2.3. Translation and Cross-Cultural Adaptation of the PASIPD Scale

Upon obtaining authorization from the PASIPD developers, the translation of the English version was conducted following international guidelines for cross-cultural adaptation of self-administered questionnaires [26].

Two native Arabic speakers with excellent command of the English language independently translated the scale from English into Arabic. The two Arabic versions were then reviewed together by the translators to identify any discrepancies, and only one version was retained. Two Saudi university professors, who were born and raised in the United States, conducted independent and blinded back translations of the predetermined Arabic version back into English. Finally, eight physical education and adapted physical activity experts, who were Saudi public university professors and alumni of American or British colleges, were selected. These experts reviewed the translated scale and provided feedback on its face and content validity. All comments and suggestions from the experts were then incorporated, and the final version of the translated scale was completed and prepared for pilot testing. The Arabic translation of the scale is provided in the supplementary materials.

#### Pilot Test

A preliminary investigation was conducted on a sample of 49 individuals with physical disabilities residing in Saudi Arabia’s eastern province. The study sample comprised 25 male participants (51.1%) and 24 female participants (48.9%) aged between 19 and 57 years old, with a mean age of 37.04 years old and a standard deviation of 9.98 years. The objective of the pilot study was to assess the test–retest reproducibility of the prefinal version of the PASIPD-AR by administering it to the same participants with a three-week interval (see Figure 1). During this phase, participants were instructed to record, in the left-hand margin next to each question, any difficulties they encountered in comprehending the questionnaire’s various components. The authors considered these comments, and the response rate to the new test was 100 percent.

### 2.4. Validity

To assess the accuracy of the PASIPD-AR, three distinct measures of validity were considered: face validity, content validity, and construct validity.

### 2.5. Face Validity and Content Validity

The concept of face validity is predicated on the notion that the items on a questionnaire have an apparent capacity to assess the intended concept. Content validity, also known as expert evaluation, is a technique used to determine the suitability of questionnaire items by assessing how well an instrument covers the various aspects of the construct under investigation. In the case of the current study, this method involved experts evaluating the logical relationship between the items and the measurement of PA levels of the participants with physical disabilities. To assess the face and content validity of the PASIPD-AR, a panel of eight experts was selected. This panel consisted of four specialists in the field of sports and physical education, as well as four specialists in adaptive physical activity. These experts were recruited from the pool of university academics at public universities in Saudi Arabia. This selection was based on the candidates’ specific areas of knowledge, research interests, and previous experience in activities related to the evaluation of measurement scales and content validity. Based on their extensive knowledge of PA theories, the experts evaluated the face validity of the PASIPD-AR by assessing its ability to accurately measured PA levels in individuals with physical disabilities. They also evaluated the content validity of the scale and its items, particularly in terms of their ability to measure PA levels in Arabic-speaking participants.

### 2.6. Construct Validity

According to Safipour et al. [23], construct validity places a strong emphasis on logical analysis and the testing of predicted relationships based on theoretical considerations. It is a comprehensive concept that includes various forms of validity. Specifically, when a measure exhibit construct validity, it will also exhibit content validity [27]. The current investigation employed confirmatory factor analysis (CFA) to estimate the factor loading of variables.

Before conducting the CFA, the Kaiser–Meyer–Olkin (KMO) measure and Bartlett’s Test and total variance explained were computed. The KMO measure of sampling adequacy (MSA) is a statistical measure used to assess the extent to which the observed variables in a study are influenced by underlying factors, indicating the proportion of variation that can be attributed to these factors. The statistical analysis software IBM SPSS 26.0 was used for the calculations. The factor analysis function was employed, with the selection of the correlation matrix option in descriptors for KMO and Bartlett’s Test of sphericity. The Varimax method was chosen for rotation, and the anti-image matrices were utilized to calculate the MSA for individual variables [28]. Previous research has indicated that a KMO measure above 0.5 is considered acceptable for conducting factor analysis. However, a KMO value exceeding 0.8 is considered highly appropriate for this analytical technique. It is typically recommended that the cumulative proportion of explained variance (also referred to as total variance explained) falls within the range of 50–90% for factor analysis.

Subsequently, CFA models inside the standard error of measurement (SEM) framework are used to assess the effect of measurement error on the model, validate a multifactor model, and determine the group factors’ influence [29]. AMOS 26.0 was used for CFA. Several statistical metrics were employed to assess the model’s fitness, including CMIN (chi-square value), which was used to determine the statistical significance of the observed variables in relation to the expected outcomes. The measure of interest is the minimum discrepancy divided by its degrees of freedom (CMIN/DF), which signifies a satisfactory fit for values ≤ 3 [30] and a reasonable fit for values ≤ 5 [31]. Additionally, the root means square error of approximation (RMSEA) is used to assess the discrepancy between the observed covariance matrix per degree of freedom and the predicted covariance matrix [32]. According to MacCallum et al. [33], RMSEA values exceeding 0.1 are deemed to be indicative of poor model fit. Values between 0.08 and 0.1 are considered borderline, suggesting a somewhat acceptable fit; values ranging from 0.05 to 0.08 are indicative of a reasonably good fit; values equal to or less than 0.05 are considered excellent.

Additionally, the root mean square (RMR) is calculated as the average absolute value of the covariance residuals. The lower limit of this variable is fixed at zero, while the upper limit is variable and contingent upon the scale of the measured variables. A model exhibiting a smaller deviation from an RMR value of 0 suggests a greater degree of adequacy. Additionally, the Akaike information criterion (AIC) is employed to evaluate the quality of fit for a given model. The chi-square value of the model is considered and adjusted to accommodate the intricacy of the model. In this specific instance, there is a lack of a defined threshold, such as 0.90. On the contrary, the measure is employed for comparing models, whereby a lower value is indicative of a more favorable match. Therefore, the AIC serves as a metric for quantifying the discrepancy between the covariance matrices derived from the model and those observed in empirical data [23].

According to Uedufy [34], the inclusion of reference comparisons is a crucial aspect of the parameters utilized for the interpretation of model-fitting outcomes in AMOS. The models utilized in the analyses are automatically fitted, encompassing numerous indices that demonstrate model adequacy, including the normed fit index (NFI), the relative fit index (RFI), the incremental fit index (IFI), the Tucker–Lewis coefficient (TLI), and the comparative fit index (CFI). All these indices truncate values between 0 and 1, and a value of 1 represents a perfect fit, although models with a value less than 0.9 can generally be improved greatly [35]. The CFI is the most used index for evaluating model fit, and a value of 0.95 or higher is considered to indicate excellent data fit [36].

### 2.7. Reliability

The notion of reliability is concerned with the extent to which a specific measurement demonstrates consistency. There are other conceptualizations of this phenomenon, including the test–retest approach and the internal consistency method [37].

The test–retest correlation analysis is performed to evaluate the association between the two sets of scores. A weak positive correlation is typically defined as a coefficient ranging from 0.1 to 0.3, while a moderate positive correlation is characterized by a coefficient ranging from 0.3 to 0.5. A high positive correlation, on the other hand, is indicated by a coefficient ranging from 0.5 to 1.0.

The intraclass correlation coefficient (ICC) for test–retest reliability is determined using a two-way mixed-effects model with absolute agreement, and a 95% confidence interval is constructed. According to Koo and Li [38], an ICC value above 0.90 is categorized as “excellent” reliability. A value between 0.75 and 0.90 is considered “good” dependability, while a value between 0.50 and 0.75 is categorized as “moderate” reliability. Finally, an ICC value below 0.50 is considered to have “poor” reliability. Additionally, the precision of the reliability results was assessed using the SEM, which was calculated as the standard deviation of the difference score divided by the square root of 2 [39]. The minimum detectable change (MDC) was determined by multiplying the SEM by the square root of 2 and then multiplying it by 1.96, corresponding to a 95% confidence level. This approach aligns with the recommendations made in recent validations of other questionnaires [40].

Internal consistency reliability pertains to the extent of consistency or uniformity within a scale, particularly in relation to how effectively the items on the scale measure the identical underlying construct. The assessment of internal consistency was performed by employing Cronbach’s alpha coefficient, which is calculated based on the mean correlation among items and the total number of items included in the scale. The variable alpha denotes the mean value of all possible split-half correlations within a set of elements. According to Hajjar [41], an item is deemed reliable if its Cronbach’s alpha score exceeds 0.6. Additionally, an item is regarded as acceptable if its Cronbach’s alpha score falls within the range of 0.6–0.8. Furthermore, a corrected item-total correlation larger than 0.3 is indicative of reliability.

The assessment of internal consistency also involved the utilization of the split-half process. Phelan and Wren [42] explained that split-half reliability can be categorized as a subtype of internal consistency reliability. One fundamental premise underlying this technique is that the two sections of the research instrument are expected to provide comparable true scores and error variances. A correlation analysis, namely Pearson’s r or Spearman’s rho, is conducted to examine the relationship between the two parts of the instrument. Subsequently, the coefficients are inputted into the Spearman–Brown formula to derive the split-half reliability coefficient. According to Faremi [43], a Guttman split-half coefficient ranging from 0.80 to 0.90 is commonly seen as indicative of a good level of reliability for research instruments.

The Guttman split-half correlation is a statistical model that offers an alternative approach, as it does not necessitate the equality of the two components of the instrument. Guttman [44] proposed a series of six reliability coefficients, which were further elaborated upon by Revelle and Zinbarg [45]. These coefficients, denoted as L1 to L6, were introduced as a means of evaluating the reliability of measurements. Specifically, Guttman [44] suggested the utilization of L4 for measuring the dependability of half the sample. It is important to mention that L3 is synonymous with Cronbach’s alpha, and Coefficient alpha reflects the mean of the split-half boundaries [27,46]. To evaluate the internal consistency reliability of the PASIPD-AR, a translated version of the questionnaire was administered to a sample of 206 individuals with physical disabilities. The participants in this study were randomly selected from three regions within Saudi Arabia.

## 3. Results

### 3.1. Participants Characteristics

Table 1 presents a comprehensive breakdown of the participants, categorized by various sub-groups including gender, marital status, education level, self-rated health, self-rated physical activity, and the utilization of mobility assistance equipment. The table provides both the absolute number and the corresponding percentage for each sub-group. The variables of age, weight, height, BMI, and PASIPD-AR score are also included in the table.

### 3.2. Validation

#### Face Validity and Content Validity

All the experts who evaluated the face validity of the PASIPD-AR scale unanimously verified that this instrument has a high level of face validity and is suitable for assessing PA levels in individuals with physical disabilities. The PASIPD-AR scale was also deemed to have excellent content validity in its ability to assess the PA levels of the participants. The above means that the PASIPD-AR can be utilized by individuals with physical disabilities in Saudi Arabia to accurately measure their levels of PA. This is because the original English version of the scale was specifically designed for usage among such participants, and as a result, both the original and translated versions of the scale contain items that can be easily understood by participants.

After conducting a thorough examination of the instrument and its constituent items, the experts were instructed to assign a score to each item separately (1, not relevant; 2, somewhat relevant; 3, quite relevant; 4, highly relevant). The relevance rating was then recoded as 1 (relevance scale of 3–4) or 0 (relevance scale of 1–2). The content validity index (CVI) for item (I-CVI), the average of the I-CVI scores for all items on the scale (S-CVI/Ave), and the proportion of items on the scale that were assigned a relevance scale of 3 or 4 by the eight experts (S-CVI/UA) were calculated as described by Yusoff [47]. Values relative to I-CVI were between 0.875 and 1, S-CVI/Ave = 0.981, and S-CVI/UA = 0.846, indicating that the PASIPD-AR achieved a satisfactory level of content validity [48].

The findings indicate that the scale and its constituent items were perceived as clear by the participants, who did not encounter any challenges in comprehending the vocabulary, concepts, and items on the scale. Furthermore, the above-mentioned experts verified that the scale can be used to accurately assess the PA levels of individuals with physical disabilities by evaluating their engagement in sports and leisure time activities, home tasks, and occupational activity.

### 3.3. Construct Validity

The findings from the KMO and Bartlett’s test revealed that the determinant of the PASIPD-AR is less than 0.001. The KMO value of the scale was found to be 0.764, which is near the desired threshold of 0.8. Furthermore, the specific values ranged from 0.672 to 0.86, suggesting that all items within the scale are suitable for factor analysis. The findings from the factor analysis revealed that the total variance explained accounted for 61.62%, which is above the threshold of 50%, and is considered sufficient for conducting factor analyses. The specific factor loadings for factors ranged from 0.583 to 0.889 (Table 2).

A second-order CFA was then performed. Heywood instances are presented in the five-factor model, suggesting that this solution is not admissible [49]. Following an extensive examination of the available data and subsequent adjustments, a revised conceptual framework was proposed using a four-factor model. Notably, Factor 3, pertaining to vigorous sport and recreational activity, and Factor 4, relating to light and moderate sport and recreational activity, were consolidated into a novel factor: *Sports and recreational activity*. The newly introduced factor was positioned third, whilst Factor 5 was relegated to fourth position. The chi-square goodness of fit was computed; the result indicated that the model did not fit the data well (*N* = 206, df = 50; chi-square 88.162, *p* = 0.001). However, the other indices revealed a good fit of data: CMIN/df = 1.763, RMR = 0.006, GFI = 0.937, CFI = 0.945, RMSEA = 0.061, and AIC = 144.162. The range of change in each variable that could be attributed to changes in its constituent items ranged from 0.49 to 1.93, with VIF values ranging from 1.236 to 2.282, indicating modest multicollinearity. The CFA analysis also shows that the change in the PASIPD-AR caused by a one-standard-deviation unit shift was 0.728 for Factor 1, 0.611 for Factor 2, 0.883 for Factor 3, and 0.73 for Factor 4 indicating moderate influence of Factor 2 and significant influence of the rest (Table 3).

### 3.4. Reliability

The stability of the entire scale was assessed through the utilization of the Spearman correlation coefficient, as demonstrated in previous studies [50,51]. The Spearman’s correlation coefficient for the entire scale was determined to be 0.97, indicating a strong positive association between the items. The coefficient values exhibited a specific range of 0.701–0.981, as indicated in Table 4. The ICC (3,1) demonstrated excellent reliability for both the PASIPD-AR scale and its four constituent subscales. The relative values were as follows: 0.973 (0.952–0.985), 0.981 (0.967–0.99), 0.933 (0.881–0.962), 0.981 (0.966–0.989), and 0.997 (0.994–0.998), respectively. The SEM and minimum detectable change at the 95% confidence level (MDC95) were as follows: 0.368–1.019 for the PASIPD-AR scale, 0.221–0.611 for the home repair, lawn, and garden work factor, 0.724–2.007 for the housework factor, 0.265–0.733 for the sports and recreation factor, and 0.262–0.726 for the occupation component.

The internal consistency was assessed using Cronbach’s alpha, yielding a coefficient of 0.694. The individual factors, including Factor 1, 2, 3, and 4, exhibited corresponding values of 0.580, 0.581, 0.619, and 0.42, respectively (Table 3). The Cronbach’s alpha coefficient was found to vary between 0.653 and 0.726 when each item of the PASIPD-AR was removed separately, as reported in other studies [27,50,51,52]. Furthermore, the Spearman–Brown coefficient and the Guttman split-half coefficient yielded values of 0.709 and 0.693, respectively. Additionally, Guttman’s bound lambda values (lambda 1–6) exhibited a range of 0.636 to 0.731, as presented in Table 4.

## 4. Discussion

Several PA scales, including the Global Physical Activity Questionnaire (GPAQ) [53] and the International Physical Activity Questionnaire (IPAQ) [54,55], have been created and approved for use in the general population. The Physical Activity Recall Assessment for People with Spinal Cord Injury (PARA-SCI) [56,57], the Physical Activity Inventory for Patients with Spinal Cord Injury (PAI-SCI) [58], the Physical Activity and Disability Survey (PADS) [59], and the PASIPD [18] represent specific scales developed to assess PA levels in specific sub-groups [60]. As the PASIPD is a scale intended to evaluate PA in all people with physical disabilities, it was selected for use in the current study as it represents the most promising self-reporting tool for gauging the level of PA [21].

In contrast to the original English version of the PASIPD, which establishes the presence of five latent factors, the statistical analysis conducted in the current study revealed the existence of only four latent factors. The PASIPD-AR consolidated the factors related to vigorous sports and leisure activities and light and moderate sports and leisure activities into a single factor: Sports and leisure activities. While adjustments were made to items 2–4 and 10 to better suit the socio-cultural considerations of the Saudi context, the fundamental objectives and scoring criteria for all items remained unaltered in the PASIPD-AR. The sole modification that occurred pertained to the latent factors. Specifically, in the PASIPD-AR, the new factor produced from Factors 3 and 4 in the original English version is positioned in third place, whereas Factor 5 is demoted to fourth place. Furthermore, considering that the evaluation of the questionnaire entails a singular computation for each item, there will be no alteration in the overall PASIPD score in the Arabic version. However, in the process of decision-making in relation to validity and reliability, it is imperative to consider the individual score assigned to each factor, while also considering the revised arrangement and composition of these factors. The composite score of the novel factor is derived by aggregating the individual scores of its constituent items. The results of our study were consistent with the findings of Ulas et al. [61], indicating that the Turkish version of the PASIPD also identified four latent variables. Specifically, the sport and recreation aspects, which were originally categorized as two independent factors, were combined into a single factor.

One notable advantage of the current study is the utilization of diverse methodologies for data analysis, which mutually reinforced one another and facilitated a more holistic comprehension of the magnitude and intricacies involved in the translation process. The translation of the scale was conducted under the guidance of experts, and subsequently, a pilot test was administered to a sample of 49 individuals with physical disabilities, consisting of 25 males and 24 females. The findings indicate that the PASIPD-AR scale possesses good face, content, and construct validity. The face and content validity of the PASIPD-AR were assessed by a team of experts who deemed that the scale and its individual items (vocabulary, concepts, and questions) would be clearly understood by the participants. The experts also confirmed that the PASIPD-AR scale can be used to accurately evaluate the PA levels of individuals with physical disabilities by assessing their involvement in sports and leisure activities, household tasks, and occupational activities. The findings pertaining to the content validity index for item (I-CVI) ranged from 0.87 to 1. The average I-CVI for all items on the scale was 0.98. Furthermore, the proportion of items on the scale that were awarded a relevance rating of 3 or 4 by the eight experts was 0.84. These results meet the valid minimum standards and suggest that the PASIPD-AR demonstrates a satisfactory level of content validity, as per Lynn’s [48] criteria. According to Lankhorst et al. [21], the original edition of the PASIPD has strong validity and significant associations with a variety of other scales. In their systematic review and analysis of the measurement properties of instruments that assess PA in wheelchair users, these authors asserted that, based on the evaluation of methodology and outcomes, the most robust evidence synthesis for the PASIPD indicates high levels of positive evidence for content and structural validity. However, there was significant negative evidence for criterion validity and modest negative evidence for hypothesis testing. Furthermore, there was considerable negative evidence for internal consistency.

Assessing the construct validity of the PASIPD-AR scale was achieved by using retrospective CFA with a sample size of 206 individuals with physical disabilities. According to Jordan and Hoefer [27], construct validity is widely regarded as the most robust type of validity and encompasses other types of validity. Therefore, it can be assumed that a research instrument that demonstrates construct validity also exhibits content validity. Prior to completing the CFA, an assessment was conducted to determine the suitability of the factor model. The findings indicated that the KMO value of the scale was 0.76, which approximates the desired threshold of 0.8. The observed values exhibited a range between 0.67 and 0.86. Bartlett’s test of sphericity yielded a statistically significant result (*p* < 0.001), providing sufficient evidence to reject the null hypothesis and suggest an acceptable link among the variables. The total variance explained results indicated that the total variance explained accounted for 61.62% of the results. This percentage is deemed sufficient for the purpose of conducting factor analyses, as stated by Rezaee and Jafari [62]. To assess the model, typical methods of maximum likelihood estimation were employed [63]. All the model-fit indices indicate that the CFA four-factor model fits well. Specifically, the CMIN/df value is less than 3.0, and the GFI and CFI values are greater than 0.90, as recommended by Bagozzi and Yi [64]. Additionally, the RMSEA value is less than 0.08, as suggested by Browne and Cudeck [65]. Hence, it can be inferred that the model effectively conforms to the data and is therefore capable of elucidating the study’s hypotheses.

The reliability of the PASIPD-AR scale was assessed using four distinct methods: (i) test–retest reliability, (ii) Cronbach’s alpha, (iii) the Spearman–Brown coefficient, and (v) Guttman’s split-half reliability. The test–retest reliability of the scale was assessed by administering it to a sample of 49 participants with physical disabilities on two occasions, with a three-week interval between administrations. The Spearman’s correlation coefficient for the entire scale was found to be 0.84, indicating a strong positive correlation between the items. The coefficient values for the items fell within the range of 0.70 to 0.98, which suggests that there is a high level of test–retest reliability for all the items. The ICC also exhibited excellent reliability for the PASIPD-AR scale and its four constituent subscales. The relative values ranged from 0.933 to 0.997, indicating that the reduction in SEM and MDC to be disregarded in future studies [39].

Nevertheless, the translated scale’s reliability, as assessed by Cronbach’s alpha, was moderately acceptable, with a value of 0.69. The factor values ranged from 0.42 to 0.61, indicating a moderate level of reliability, while the internal consistency values ranged from 0.65 to 0.72. It is suggested that an item be retained in a scale if its Cronbach’s alpha coefficient is 0.70 or above [27,52]. In the original English version of the PASIPD, the reliability coefficient, as measured by Cronbach’s alpha, was found to be 0.6, with individual factor values for each of the five variables ranging from 0.37 to 0.65. According to Washburn et al. [18], the identified low-to-moderate level of internal consistency can be attributed to the limited number of items within each factor.

The measurement of split-half reliability was conducted using both the Spearman–Brown and Guttman’s split-half reliability methods. The Spearman–Brown coefficient was 0.709, which was moderately under the recommended value of 0.80 or higher for adequate reliability [28]. The Guttman split-half coefficient yielded a value of 0.693, suggesting also a moderate internal consistency dependability. The present approach is a modification of the Spearman–Brown coefficient that does not necessitate equal variance between the two split versions. The Guttman’s lower limit lambda (lambda 1–6) exhibited moderate values, and the reliability estimate of the items was determined by selecting the highest value from the lower bound Guttman split-half bound, in accordance with Guttman’s recommendations [28].

The experts’ evaluation and assessment of the study’s findings show that the PASIPD-AR scale is considered valid for evaluating the level of PA in people with physical disabilities in the Saudi Arabian context.

Furthermore, no linguistic issues that would affect the intelligibility of the concepts were found during the translation process. However, it is crucial to recognize some limitations. First, it is imperative to emphasize that the PASIPD-AR scale is only appropriate for use among adult Arabic-speaking patients with physical disabilities. Before being implemented, its application in various disability contexts must be validated. It’s also crucial to remember that all results were derived from self-reported data provided by the participants themselves, which raises the possibility of bias that cannot be ignored. Finally, the potential use of the PASIPD-AR may be associated with instances of Heywood cases, primarily due to the diminished number of items within Factor 4 (specifically, two items). The term “Heywood case” pertains to a factorial solution wherein the estimation of error variance yields a negative value, leading to the production of a matrix that is non-positive definite. The utilization of the PASIPD-AR may give rise to this issue, particularly when outliers are present and/or when the sample size is limited. Hence, to ensure the attainment of dependable and uniform estimations, it is advisable to have a minimum sample size exceeding 150 and to eliminate any outliers before conducting any analysis [66].

## 5. Conclusions

The assessment of the PASIPD-AR demonstrated a satisfactory level of reliability and validity, making it suitable for use in the Saudi Arabian context. Experts and CFA were employed to evaluate the scale’s face, content, and construct validity. Reliability was assessed through four independent approaches: test–retest reliability, Cronbach’s alpha, the Spearman–Brown coefficient, and Guttman’s split-half dependability. Additionally, the findings revealed that, in contrast to the original English version, which identified five underlying factors, the PASIPD-AR detected only four latent factors. The factors related to intense sports and recreational activities, as well as gentle and moderate sports and recreational activities, were combined into a single component referred to as “sports and leisure activities”.

This scale can be utilized by physiotherapists and doctors to assess the PA level of individuals cost-effectively and straightforwardly with physical disabilities. While further research is required to expand upon our initial findings, we assert that the current study provides impetus for future investigations pertaining to our comprehensive study, specifically within populations with varying disabilities. Subsequent research endeavors should aim to evaluate the construct validity of the scale through a comparative analysis of its findings with those obtained through direct assessment methods. Additionally, it is advisable for future research endeavors to undertake comparative analyses between the outcomes obtained from the scale and those derived from alternative scales, namely the Global Physical Activity Questionnaire, the International Physical Activity Questionnaire, the Physical Activity Recall Assessment for People with Spinal Cord Injury, and the Physical Activity and Disability Survey.

## Figures and Tables

**Table 1:** Descriptive characteristics of participants identified with physical disabilities (*N* = 206).

	*N* (%)	Age	Weight (kg)	Height (cm)	BMI (kg/m^2^)	PASIPD-AR
Gender						
Male	134 (65.0%)	39.66 ± 12.22	73.11 ± 24.00	159.49 ± 23.66	33.90 ± 35.38	8.48 ± 9.42
Female	72 (35.0%)	39.38 ± 12.15	70.32 ± 24.93	150.11 ± 22.13	36.00 ± 34.76	8.87 ± 9.25
Marital status						
Single	80 (38.8%)	30.21 ± 9.71	59.90 ± 17.41	155.45 ± 21.25	27.07 ± 20.18	7.24 ± 7.82
Married	101(49%)	45.05 ± 9.19	78.54 ± 22.63	157.98 ± 23.50	38.00 ± 41.77	9.17 ± 10.18
Divorced	16 (7.8%)	46.00 ± 10.88	82.25 ± 36.71	146.75 ± 36.61	50.72 ± 48.56	12.31 ± 10.90
Widowed	4 (1.9%)	56.75 ± 8.96	76.25 ± 9.46	157.50 ± 10.21	31.07 ± 5.71	14.52 ± 7.17
Do not want to respond	5 (2.4%)	44.00 ± 10.51	102.80 ± 27.10	162.00 ± 3.81	39.08 ± 9.49	2.98 ± 4.23
Education level						
Primary school degree	44 (21.4%)	44.89 ± 12.89	68.27 ± 22.29	150.89 ± 26.88	35.23 ± 29.99	8.27 ± 9.04
Middle school degree	23 (11.2%)	44.87 ± 11.53	81.57 ± 31.83	153.17 ± 33.72	51.48 ± 70.25	6.89 ± 7.38
High school degree	76 (36.9%)	36.71 ± 11.68	71.12 ± 23.49	159.80 ± 18.27	30.60 ± 27.74	9.08 ± 9.39
University degree	57 (27.7%)	37.67 ± 11.16	72.68 ± 24.17	157.82 ± 18.45	30.46 ± 13.87	9.06 ± 10.06
Postgraduate degree	6 (2.9%)	34.33 ± 6.09	72.00 ± 10.75	146.17 ± 44.67	56.37 ± 70.92	7.69 ± 12.65
Self-rated health						
Poor	26 (12.6%)	44.65 ± 12.88	75.73 ± 29.38	156.23 ± 25.28	33.65 ± 21.26	6.57 ± 7.42
Good	139 (67.5%)	39.50 ± 11.93	71.14 ± 22.90	156.23 ± 23.97	34.91 ± 38.60	8.68 ± 9.55
Excellent	41 (19.9%)	36.56 ± 11.71	73.22 ± 25.79	156.15 ± 21.24	34.33 ± 29.82	9.69 ± 9.72
Self-rated physical activity						
Not active at all	61 (29.6%)	42.38 ± 13.22	77.33 ± 31.97	154.79 ± 25.41	37.63 ± 40.65	8.87 ± 8.96
Moderately active	128 (62.1%)	38.54 ± 11.50	70.67 ± 20.45	157.13 ± 21.45	32.89 ± 30.91	8.45 ± 9.51
Active/extremely active	17 (8.3%)	37.18 ± 12.08	64.53 ± 15.37	154.41 ± 31.51	36.97 ± 43.98	8.95 ± 9.95
Use of mobility assistive device						
Independent	42 (20.4%)	36.88 ± 11.25	71.10 ± 21.98	155.52 ± 24.91	35.19 ± 33.02	10.31 ± 9.53
Wheelchair	120 (58.3%)	39.96 ± 12.84	71.86 ± 26.20	155.12 ± 24.62	34.51 ± 32.00	8.63 ± 9.67
Crutches	24 (11.7%)	41.38 ± 12.58	70.17 ± 19.54	154.83 ± 23.66	39.39 ± 60.21	4.82 ± 4.24
Walking sticks	20 (9.7%)	40.65 ± 8.77	78.35 ± 22.82	165.90 ± 6.60	28.50 ± 8.22	9.54 ± 10.63

**Table 2:** Item correlation with total score factor loading, Eigenvalues, and percentage of variance explained for PASIPD-AR.

Correlation with Total Score		Factor Loading			
		Factor 1: Home Repair Lawn and Garden Work	Factor 2: Housework	Factor 3: Sports and Recreation	Factor 4: Occupation
Walk and wheel push outside home (not for exercise)	0.646 **	-	-	-	0.750
Light sport and recreation	0.582 **	-	-	0.603	-
Moderate sport and recreation	0.541 **	-	-	0.628	-
Strenuous sport and recreation	0.418 **	-	-	0.720	-
Exercise to increase muscular strength	0.468 **	-	-	0.618	-
Light housework	0.544 **	-	0.889	-	-
Heavy housework	0.614 **	-	0.649	-	-
Home repair	0.451 **	0.600	-	-	-
Lawn work and yard care	0.482 **	0.822	-	-	-
Moderate sports and recreational activities	0.401 **	0.778	-	-	-
Outdoor garden work	0.481 **	-	0.583	-	-
Paid employment/volunteering	0.612 **	-	-	-	0.617
Eigenvalues	-	2.442	1.711	1.956	1.285
% Variance	-	20.353	14.261	16.301	10.705
Cumulative % variance	-	20.353	34.614	50.915	61.621

Note: ** *p* < 0.001.

**Table 3:** Item value, Cronbach’s alpha, Beta weights, and variance inflation factor (VIF) for PASIPD-AR.

PASIPD-AR Items, Subscales, and Total	Four-Factors Model				
	Mean ± SD (MET h/d)	Cronbach’s Alpha	Beta Weights		VIF
			Item-Factor	Factor- PASIPD-AR	
**Factor 1: Home repair, lawn, and garden work**	1.362 ± 4.86	0.580		0.728	
Home repairs	0.484 ± 1.634		0.73		1.365
Lawn work or yard care	0.472 ± 1.965		0.62		2.272
Outdoor gardening	0.406 ± 1.820		0.70		2.282
**Factor 2: Housework**	2.612 ± 4.918	0.581		0.611	
Light housework	0.640 ± 1.343		0.73		1.910
Heavy housework or chores	0.906 ± 2.639		1.81		2.269
Caring for another person	1.065 ± 1.966		0.53		1.236
**Factor 3: Light to vigorous sport and recreational activity**	3.99 ± 9.7	0.619		0.883	
Strenuous sports and recreational activities	1.111 ± 3.757		0.73		1.409
Exercise to increase muscle strength and endurance	1.405 ± 3.671		0.67		1.539
Light sports or recreational activities	0.617 ± 1.634		0.70		1.485
Moderate sports and recreational activities	0.859 ± 2.537		0.49		1.310
**Factor 4: Occupational and transportation activity**	5.03 ± 7.227	0.42		0.730	
Walk, wheel, push outside home.	2.754 ± 3.502		0.73		1.272
Paid employment/volunteering	2.277 ± 5.257		1.93		1.371
PASIPD-AR	12.996 ± 20.424	0.694			

**Table 4:** Reliability coefficients.

		Reliability				
Test–Retest Correlation		Internal Consistency Cronbach’s Alpha	Split-Half			
Walk and wheel push outside home (not for exercise)	0.969 **	0.726	Cronbach’s Alpha	Part 1	Value	0.684
					*N* of Items	6 a
Light sport and recreation	0.956 **	0.657		Part 2	Value	0.444
Moderate sport and recreation	0.981 **	0.664			*N* of Items	6 b
Strenuous sport and recreation	0.944 **	0.681		Total *N* of Items		12
Exercise to increase muscular strength	0.977 **	0.674	Correlation Between Forms			0.55
Light housework	0.902 **	0.667	Spearman–Brown Coefficient		Equal Length	0.709
Heavy house work	0.701 **	0.653			Unequal Length	0.709
Home repair	0.920 **	0.676	Guttman Split-Half Coefficient			0.693
Lawn work and yard care	0.968 **	0.681	Lambda		1	0.636
					2	0.713
Outdoor garden work	0.835 **	0.689			3	0.694
					4	0.693
Caring for another person	0.740 **	0.683			5	0.711
					6	0.731
Paid employment/volunteering	0.969 **	0.659	a. items 3–8. b. items 2, 9–13.			

Note: ** *p* < 0.001.

## Data Availability

The data that support the findings of this study are available from the author upon reasonable request.

## Supplementary Materials

The following supporting information can be downloaded at: https://www.mdpi.com/article/10.3390/healthcare12020179/s1, The Arabic Version of the Physical Activity Scale for Individuals with Physical Disabilities in Saudi Arabia (PASIPD-AR).
