# Supplementary material for: Cross-Cultural Adaptation and Validation of the Arabic Version of the Physical Activity Scale for Individuals with Physical Disabilities in Saudi Arabia (PASIPD-AR)
Source: Healthcare (Basel). 2024 Jan 11;12(2):179. doi: 10.3390/healthcare12020179 (PMC10815588; doi:10.3390/healthcare12020179)
Supplement: Supplementary file 1 [file healthcare-12-00179-s001.zip › healthcare-2781400-supplementary.pdf]

# The Arabic Version of the Physical Activity Scale for Individuals with Physical Disabilities in Saudi Arabia (PASIPD-AR)

# النسخة العربية من مقياس النشاط البدني للأفراد ذوي الإعاقة الحركية في المملكة العربية السعودية (PASIPD-AR)

**التعليمات:**

هذا المقياس هدفه تقييم مستوى النشاط البدني والتمرين الرياضي الحالي لديك، حيث لا توجد هناك إجابات صحيحة أو خاطئة.

**الأنشطة في أوقات الفراغ**

1. خلال الأيام الـ 7 الماضية، كم مرة مارست أنشطة ترفيهية أثناء الجلوس مثل (القراءة، مشاهدة التلفاز، ألعاب الكمبيوتر، أو ممارسة الأعمال اليدوية)؟

أ. لم أقم بذلك إطلاقًا. (إذا كان هذا اختيارك، اذهب إلى السؤال رقم 2 مباشرة)

ب. نادرًا (1-2 يوم في الأسبوع)

ج. أحيانًا (3-4 يوم في الأسبوع)

د. غالبًا (5-7 يوم في الأسبوع)

كم متوسط عدد الساعات التي قضيتها خلال اليوم الواحد في ممارسة هذه الأنشطة أثناء الجلوس؟

أ. أقل من ساعة واحدة.

ب. من ساعة واحدة إلى أقل من ساعتين.

ج. من 2-4 ساعات.

د. أكثر من 4 ساعات.

2. خلال الأيام الـ 7 الماضية، كم مرة تنقلت (على قدميك أو بكرسي مُتحرك) خارج المنزل لغرض آخر غير الرياضة. على سبيل المثال، الذهاب إلى العمل أو الدراسة، التنزه، التسوق، أو المهام الأخرى؟

أ. لم أقم بذلك إطلاقًا. (إذا كان هذا اختيارك، اذهب إلى السؤال رقم 3 مباشرة)

ب. نادرًا (1-2 يوم في الأسبوع)

ج. أحيانًا (3-4 يوم في الأسبوع)

د. غالبًا (5-7 يوم في الأسبوع)

كم متوسط عدد الساعات التي قضيتها في اليوم الواحد خارج المنزل على الكرسي المُتحرك سوآءا كان دفع الكرسي من قبلك أو بمساعدة أشخاص آخرين؟

أ. أقل من ساعة واحدة.

ب. من ساعة واحدة إلى أقل من ساعتين.

ج. من 2-4 ساعات.

د. أكثر من 4 ساعات.

3. خلال الأيام الـ 7 الماضية، كم مرة شاركت في أنشطة رياضية أو ترفيهية خفيفة مثل البولينغ، الغولف باستخدام عربة الغولف، الرماية، صيد السمك، رمي السهام، البلياردو، التمرينات العلاجية (العلاج الطبيعي أو التّمدد)، أو أي أنشطة أخرى مشابهة؟

أ. لم أقم بذلك إطلاقًا. (إذا كان هذا اختيارك، اذهب إلى السؤال رقم 4 مباشرة)

ب. نادرًا (1-2 يوم في الأسبوع)

ج. أحيانًا (3-4 يوم في الأسبوع)

د. غالبًا (5-7 يوم في الأسبوع)

كم متوسط عدد الساعات التي قضيتها في اليوم الواحد في ممارسة هذه الأنشطة الرياضية الترفيهية الخفيفة؟

أ. أقل من ساعة واحدة.

ب. من ساعة واحدة إلى أقل من ساعتين.

ج. من 2-4 ساعات.

د. أكثر من 4 ساعات.

4. خلال الأيام الـ 7 الماضية، كم مرة شاركت في أنشطة رياضية أو ترفيهية متوسطة الشدة مثل تنس الطاولة الزوجي، الرقص الشعبي، دفع الكرسي من قبلك أو بمساعدة أشخاص آخرين، أو أي أنشطة أخرى مشابهة؟

أ. لم أقم بذلك إطلاقًا. (إذا كان هذا اختيارك، اذهب إلى السؤال رقم 5 مباشرة)

ب. نادرًا (1-2 يوم في الأسبوع)

ج. أحيانًا (3-4 يوم في الأسبوع)

د. غالبًا (5-7 يوم في الأسبوع)

كم متوسط عدد الساعات التي قضيتها في اليوم الواحد في ممارسة هذه الأنشطة الرياضية الترفيهية متوسطة الشدة؟

أ. أقل من ساعة واحدة.

ب. من ساعة واحدة إلى أقل من ساعتين.

ج. من 2-4 ساعات.

د. أكثر من 4 ساعات.

5. خلال الأيام الـ 7 الماضية، كم مرة شاركت في أنشطة رياضية أو ترفيهية شديدة أو عالية الشدة مثل السباق بالكرسي المُتحرك (تدريب)، الأيروبيكس، السباحة، تحريك الذراع، ركوب الدراجات (سوآءا باليد أو الرجل)، التنس الفردي، كرة السلة، المشي بالعكاز والحمالات، أو أي أنشطة أخرى مشابهة؟

أ. لم أقم بذلك إطلاقًا. (إذا كان هذا اختيارك، اذهب إلى السؤال رقم 6 مباشرة)

ب. نادرًا (1-2 يوم في الأسبوع)

ج. أحيانًا (3-4 يوم في الأسبوع)

د. غالبًا (5-7 يوم في الأسبوع)

كم متوسط عدد الساعات التي قضيتها في اليوم الواحد في ممارسة هذه الأنشطة الرياضية أو الترفيهية شديدة أو عالية الشدة؟

أ. أقل من ساعة واحدة.

ب. من ساعة واحدة إلى أقل من ساعتين.

ج. من 2-4 ساعات.

د. أكثر من 4 ساعات.

6. خلال الأيام الـ 7 الماضية، كم مرة مارست أي نوع من أنواع الرياضة الخاصة بزيادة قوة العضلات والتحمّل مثل رفع الأثقال، تمارين الضغط، تمارين السحب، الغطس، أو دفع الكرسي المتحرك؟

أ. لم أقم بذلك إطلاقًا. (إذا كان هذا اختيارك، اذهب إلى السؤال رقم 7 مباشرة)

ب. نادرًا (1-2 يوم في الأسبوع)

ج. أحيانًا (3-4 يوم في الأسبوع)

د. غالبًا (5-7 يوم في الأسبوع)

كم متوسط عدد الساعات التي قضيتها في اليوم الواحد في ممارسة هذه الرياضات الخاصة بزيادة قوة العضلات والتحمّل؟

أ. أقل من ساعة واحدة.

ب. من ساعة واحدة إلى أقل من ساعتين.

ج. من 2-4 ساعات.

د. أكثر من 4 ساعات.

**الأنشطة المنزلية**

7. خلال الأيام الـ 7 الماضية، كم مرة مارست أعمال منزلية خفيفة مثل تنظيف الغبار، مسح الأرضيات، أو غسل الصحون؟

أ. لم أقم بذلك إطلاقًا. (إذا كان هذا اختيارك، اذهب إلى السؤال رقم 8 مباشرة)

ب. نادرًا (1-2 يوم في الأسبوع)

ج. أحيانًا (3-4 يوم في الأسبوع)

د. غالبًا (5-7 يوم في الأسبوع)

كم متوسط عدد الساعات التي قضيتها في اليوم الواحد في ممارسة هذه الأعمال المنزلية الخفيفة؟

أ. أقل من ساعة واحدة.

ب. من ساعة واحدة إلى أقل من ساعتين.

ج. من 2-4 ساعات.

د. أكثر من 4 ساعات.

8. خلال الأيام الـ 7 الماضية، كم مرة مارست أعمال منزلية شاقة مثل التنظيف بالمكنسة الكهربائية، فرك الأرضيات، غسل النوافذ أو الجدران، وما إلى ذلك؟

أ. لم أقم بذلك إطلاقًا. (إذا كان هذا اختيارك، اذهب إلى السؤال رقم 9 مباشرة)

ب. نادرًا (1-2 يوم في الأسبوع)

ج. أحيانًا (3-4 يوم في الأسبوع)

د. غالبًا (5-7 يوم في الأسبوع)

كم متوسط عدد الساعات التي قضيتها في اليوم الواحد في ممارسة هذه الأعمال المنزلية الشاقة؟

أ. أقل من ساعة واحدة.

ب. من ساعة واحدة إلى أقل من ساعتين.

ج. من 2-4 ساعات.

د. أكثر من 4 ساعات.

9. خلال الأيام الـ 7 الماضية، كم مرة قمت بأعمال إصلاحات منزلية مثل النجارة، الطلاء، تأثيث، إصلاحات كهربائية، وما إلى ذلك؟

أ. لم أقم بذلك إطلاقًا. (إذا كان هذا اختيارك، اذهب إلى السؤال رقم 10 مباشرة)

ب. نادرًا (1-2 يوم في الأسبوع)

ج. أحيانًا (3-4 يوم في الأسبوع)

د. غالبًا (5-7 يوم في الأسبوع)

كم متوسط عدد الساعات التي قضيتها في اليوم الواحد في القيام بهذه الإصلاحات المنزلية؟

أ. أقل من ساعة واحدة.

ب. من ساعة واحدة إلى أقل من ساعتين.

ج. من 2-4 ساعات.

د. أكثر من 4 ساعات.

10. خلال الأيام الـ 7 الماضية، كم مرة قمت بأعمال العناية بالحديقة أو المزرعة مثل قص الزرع، إزالة أوراق الشجر، تقطيع الخشب، وما إلى ذلك؟

أ. لم أقم بذلك إطلاقًا. (إذا كان هذا اختيارك، اذهب إلى السؤال رقم 11 مباشرة)

ب. نادرًا (1-2 يوم في الأسبوع)

ج. أحيانًا (3-4 يوم في الأسبوع)

د. غالبًا (5-7 يوم في الأسبوع)

كم متوسط عدد الساعات التي قضيتها في اليوم الواحد في القيام بأعمال العناية بالحديقة أو المزرعة؟

أ. أقل من ساعة واحدة.

ب. من ساعة واحدة إلى أقل من ساعتين.

ج. من 2-4 ساعات.

د. أكثر من 4 ساعات.

11. خلال الأيام الـ 7 الماضية، كم مرة قمت بزراعة النباتات والأشجار بالحديقة الخارجية؟

أ. لم أقم بذلك إطلاقًا. (إذا كان هذا اختيارك، اذهب إلى السؤال رقم 12 مباشرة)

ب. نادرًا (1-2 يوم في الأسبوع)

ج. أحيانًا (3-4 يوم في الأسبوع)

د. غالبًا (5-7 يوم في الأسبوع)

كم متوسط عدد الساعات التي قضيتها في اليوم الواحد في القيام بأعمال العناية بزراعة النباتات والأشجار بالحديقة الخارجية؟

أ. أقل من ساعة واحدة.

ب. من ساعة واحدة إلى أقل من ساعتين.

ج. من 2-4 ساعات.

د. أكثر من 4 ساعات.

12. خلال الأيام الـ 7 الماضية، كم مرة قمت برعاية شخص آخر مثل (طفل، زوج أو زوجة، أو شخص آخر بالغ)؟

أ. لم أقم بذلك إطلاقًا. (إذا كان هذا اختيارك، اذهب إلى السؤال رقم 13 مباشرة)

ب. نادرًا (1-2 يوم في الأسبوع)

ج. أحيانًا (3-4 يوم في الأسبوع)

د. غالبًا (5-7 يوم في الأسبوع)

كم متوسط عدد الساعات التي قضيتها في اليوم الواحد في رعاية شخص آخر؟

أ. أقل من ساعة واحدة.

ب. من ساعة واحدة إلى أقل من ساعتين.

ج. من 2-4 ساعات.

د. أكثر من 4 ساعات.

**أنشطة متعلقة بالوظيفة**

13. خلال الأيام الـ 7 الماضية، كم مرة قمت بالعمل من أجل كسب المال أو كمتطوع؟ (استبعد الأعمال التي تتضمن الجلوس مع حركات خفيفة للذراعين مثل أعمال المكتب البسيطة، أعمال الحاسوب، أعمال التجميع، قيادة حافلة أو شاحنة، وما إلى ذلك).

أ. لم أقم بذلك إطلاقًا. (إذا كان هذا اختيارك، لا تُجب على السؤال التالي)

ب. نادرًا (1-2 يوم في الأسبوع)

ج. أحيانًا (3-4 يوم في الأسبوع)

د. غالبًا (5-7 يوم في الأسبوع)

كم متوسط عدد الساعات التي قضيتها في اليوم الواحد في العمل من أجل كسب المال أو كمتطوع؟

أ. أقل من ساعة واحدة.

ب. من ساعة واحدة إلى أقل من ساعتين.

ج. من 2-4 ساعات.

د. أكثر من 4 ساعات.

**كيفية حساب مؤشر النشاط البدني العام**

1-مضاعفات البنود

1. غير محسوبة
2. 2.5
3. 3.0
4. 4.0
5. 8.0
6. 5.5
7. 1.5
8. 4.0
9. 4.0
10. 4.0
11. 4.0
12. 1.5
13. 2.5

2. حساب متوسط الساعات في اليوم للبنود 2 - 12

| الفئة | المبلغ عنها (ساعة/يوم) | المتوسط (ساعة/يوم) |
|---|---|---|
| نادرًا<br>(من يوم إلى يومين) | <1 | 0.11 |
| | 1–2 | 0.32 |
| | 2–4 | 0.64 |
| | >4 | 1.07 |
| أحيانًا<br>(3-4 أيام) | <1 | 0.25 |
| | 1–2 | 0.75 |
| | 2–4 | 1.50 |
| | >4 | 2.50 |
| في كثير من الأحيان<br>(5-7 أيام) | <1 | 0.43 |
| | 1–2 | 1.29 |
| | 2–4 | 2.57 |
| | >4 | 4.29 |

3. حساب متوسط الساعات في اليوم للبند 13

| الفئة | المبلغ عنه (ساعة/يوم) | المتوسط (ساعة/يوم) |
|---|---|---|
| نادرًا<br>(من يوم إلى يومين) | <1 | 0.12 |
| | 4-1 | 0.64 |
| | 8-5 | 1.39 |
| | >8 | 1.93 |
| أحيانًا<br>(3-4 أيام) | <1 | 0.28 |
| | 4-1 | 1.5 |
| | 8-5 | 3.11 |
| | >8 | 4.5 |
| | <1 | 0.49 |

| | | |
|---|---|---|
| في كثير من الأحيان (5-7 أيام) | 1-4 | 2.57 |
| | 5-8 | 5.57 |
| | 8< | 7.71 |

**مؤشر النشاط البدني = مجموع مضاعفات العبارات x متوسط الساعات اليومية على البنود 2-13**

**كيفية حساب مؤشر النشاط البدني الخاص بكل محور**

المحور 1: إصلاح المنزل وأعمال الحدائق (البنود 9، 10، 11)

المحور 2: الأعمال المنزلية (البنود 7، 8، 12)

المحور 3: الرياضة والترفيه (البنود 3، 4، 5، 6)

المحور 4: المهنة (البنود 2 و13)

**مؤشر النشاط البدني الخاص بالمحور = مجموع مضاعفات البنود الخاصة بالمحور x متوسط الساعات اليومية على البنود الخاصة بالمحور**
